# Interplay of space radiation and microgravity in DNA damage and DNA damage response

**DOI:** 10.1038/s41526-017-0019-7

**Published:** 2017-05-10

**Authors:** María Moreno-Villanueva, Michael Wong, Tao Lu, Ye Zhang, Honglu Wu

**Affiliations:** 1 NASA, Johnson Space Center, Houston, TX USA; 2 University of Konstanz, Konstanz, 78457 Germany; 3 Washington University School of Medicine, St. Louis, MO USA; 4 University of Houston Clear Lake, Houston, TX USA; 5 NASA Kennedy Space Center, Cape Canaveral, FL USA

## Abstract

In space, multiple unique environmental factors, particularly microgravity and space radiation, pose constant threat to the DNA integrity of living organisms. Specifically, space radiation can cause damage to DNA directly, through the interaction of charged particles with the DNA molecules themselves, or indirectly through the production of free radicals. Although organisms have evolved strategies on Earth to confront such damage, space environmental conditions, especially microgravity, can impact DNA repair resulting in accumulation of severe DNA lesions. Ultimately these lesions, namely double strand breaks, chromosome aberrations, micronucleus formation, or mutations, can increase the risk for adverse health effects, such as cancer. How spaceflight factors affect DNA damage and the DNA damage response has been investigated since the early days of the human space program. Over the years, these experiments have been conducted either in space or using ground-based analogs. This review summarizes the evidence for DNA damage induction by space radiation and/or microgravity as well as spaceflight-related impacts on the DNA damage response. The review also discusses the conflicting results from studies aimed at addressing the question of potential synergies between microgravity and radiation with regard to DNA damage and cellular repair processes. We conclude that further experiments need to be performed in the true space environment in order to address this critical question.

## Introduction

As humans on Earth, we are well protected from insults originated in deep space. However, during space travel, all living organisms are exposed to a number of unique environmental stress factors, such as space radiation and microgravity. These two factors in particular have been topics of active investigation as their potential to compromise human health has not yet been fully elucidated.

Typically encountered beyond the reach of the Earth’s magnetosphere, space radiation refers to galactic cosmic rays (GCR) and protons released from large solar particle events (SPEs). GCRs consist of approximately 87% hydrogen ions (protons) and 12% helium ions (alpha particles), with the remaining 1–2% of particles being high atomic number and energy nuclei.^1^ In low Earth orbit (LEO), astronauts are exposed mostly to protons trapped in Earth’s geomagnetic field, and some high energy GCR and SPE particles that are able to penetrate to LEO. In fact, the energy of some GCR particles is so high that it is difficult to shield from them at all using conventional materials,^2^ making exposure inside spacecraft inevitable. Notable effects of this space radiation in humans include, for instance, light flashes from retinal exposure to charged particles,^3^ reported during astronauts’ trips to the Moon^4^ and in Skylab missions,^5^ and the early onset of cataracts.^6^ Apart from direct space radiation effects, indirect damage through bystander effects, adaptive responses, and genomic instability may also occur.^7^

In addition to radiation, in LEO or during transit to the Moon or Mars, astronauts are exposed to microgravity. As many have noted before, exposure to the microgravity environment causes a range of detrimental health effects, including bone loss, cardiovascular deconditioning, and neurovestibular changes.^8, 9^ Furthermore, some of these health effects have been observed in studies using ground-based analogs that simulate the microgravity condition.^10^

At the molecular level, radiation and/or microgravity are known to negatively impact DNA integrity. To counteract DNA damage, cells have developed specific mechanisms that locate and repair DNA lesions. These mechanisms consist of a network of cellular proteins involved in DNA damage response (DDR) pathways, such as cell cycle regulation, DNA repair, and apoptosis. Generally, cells are able to accommodate moderate DNA damage through these different repair processes; however, space conditions, especially the lack of gravity may adversely affect the DNA repair process leading to accumulation of DNA injuries.

Although space radiation and microgravity are two major environmental stressors encountered in space travel, other space-related factors can present challenges to DNA integrity as well. Specifically, partial gravity experienced on the surface of the Moon or Mars, psychological stress due to confined space or loneliness in long duration missions, possible toxic chemical compounds in the spacecraft, and lunar or Martian regolith inhaled into the lung might also negatively impact astronauts’ health.

Given the importance of understanding the consequences of the space environment on human physiology, this review focuses on the interplay of space radiation and microgravity in the DDR. Relevant studies addressing the question of potential synergies between radiation and microgravity are also discussed. Additionally, investigations of DNA damage induced in space are reviewed. Recent evidence for such effects (Tables 1 and 2) is summarized below.

**Table 1:** Summary of results related to residual DNA damage and DDR from experiments conducted in simulated microgravity

Ref.	Environment	Experimental conditions animal/cellular model	DDR	
	R, μG, R + μG		Residual damage/DNA repair activity	Synergy*
Risin and Pellis^18^	Simulated microgravity RWV + radiation	Peripheral blood mononuclear cells were exposed to ^137^Cs γ rays at a dose rate of 15 Gy/min.	Inhibition of radiation-induced apoptosis	Yes
Mosesso et al.^23^	Clinostat + radiation	Human lymphocytes were exposed to 1.5 Gy X-ray.	Increased yield of chromosome aberrations	Yes
Manti et al.^24^	Simulated microgravity RWV + radiation	Human peripheral blood lymphocytes were exposed to 60 MeV protons or 250 kVp X-rays in the dose range 0–6 Gy, and allowed to repair DNA damage for 24 h.	No differences in radiation-induced chromosome aberrations	No
Canova et al.^25^	Simulated microgravit γ RWV + radiation	Lymphoblastoid TK6 cells were irradiated with γ rays and incubated for 24 h. Radiation doses ranged from 1–4 Gy at dose rate of 1 Gy/min from ^60^Co γ source.	Higher frequency of micronucleated cells and mutations. Significant reduction in apoptotic cells. Increased number of cells in G1-phase	Yes
Dang et al.^29^	Simulated microgravity RWV + radiation	Human B lymphoblast cells were exposed to 300 MeV/u carbon ions.	Increased apoptosis and ROS	Yes
Mognato et al.^26^	Simulated microgravity RWV + radiation	Human lymphocytes were exposed to X-rays at 2.2 Gy/min or γ from ^60^Co at 1 Gy/min and incubated for 24 h. Total doses were 1, 2 and 3 Gy.	Increased mutant frequency after radiation	Yes
Mognato^28^	Simulated microgravity RWV + radiation	Human peripheral blood cells were exposed to ^137^Cs γ rays. The total dose was 5 Gy and the dose rate was 3.1 Gy/min.	Significantly higher number of γ-H2AX foci per nucleus. The fraction of foci-positive cells decreased slower after irradiation	Yes
Wang et al.^22^	Simulated microgravity 3D clinostat + radiation	Embryonic stem cells were exposed to 8 Gy γ rays and incubated for 0, 1 and 2 h post irradiation.	Increased apoptosis. Population growth inhibited.	Yes
Girardi et al.^27^	Reduced gravitational force RWV + radiation	Human peripheral lymphocytes were incubated for 4 and 24 h after 0.2 and 2 Gy γ irradiation.	Decreased number of miRNAs in response to radiation exposure	Yes
Li et al.^30^	Tail suspension + radiation	Spermatogenic cells of male Swiss Webster mice were exposed whole body to 200 MeV/u carbon ions at an entrance dose rate of 0.5 Gy/min.	Increased DNA damage. Increased apoptotic rate and the expression levels of p53 and Bax	Yes

* Synergy in terms of effect of microgravity on the repair of artificially induced DNA damage

**Table 2:** Summary of results related to residual DNA damage and DDR from experiments conducted in spaceflights

Ref.	Environment	Experimental conditions animal/cellular model	DDR	
	R, μG, R + μG		Residual damage/DNA repair activity	Synergy*
Bender et al.^39^	Gemini III mission	Cultured human lymphocytes were exposed to ^32^Pb particles in space after reaching the microgravity condition	Chromosomes deletions	Yes
Bender et al.^40^	Gemini XI mission	Cultured human lymphocytes were exposed to ^32^Pb particles in space after reaching the microgravity condition	Chromosomes deletions	No
Horneck et al.^45^	Spacelab mission IML-2	*Frozen E. coli* and *B. subtilis* bacteria and human fibroblasts were exposed to X-rays and UV before launch. Cells were thawed in space to allow repair in the microgravity environment.	Re-joining of DNA strand breaks	No
Takahashi et al.^43^	US Space Shuttle mission (Discovery; STS-91)	DNA substrate was damaged by restriction enzyme digestion in space	T4 DNA ligase activity	No
Pross et al.^47^	Radiation exposure on Shuttle Atlantis flight STS-84	Temperature dependent repair mutant rad54–3 of *S. cerevisiae* yeast was irradiated with 63Ni β particles during flight and allowed repair at different temperatures.	Double strand breaks and survival	No
Ohnishi et al.^44^	US Space Shuttle mission (Discovery, STS-91)	Damaged template DNA induced with an alkylating agent (N-methyl-N-nitroso urea; MNU).	Mutation frequency	No
Ishizaki^49^	US Space Shuttle mission (STS-95)	Human colon cancer cells were treated with bleomycin in space.	Microsatellite mutation rates	No
Wu et al.^51^	8-days Space Shuttle mission STS-103	Blood samples collected from an astronaut before and post the Shuttle mission were exposed to high doses of γ rays.	Chromosomal aberrations	No
Greco et al.^37^	Missions of different durations on the MIR and ISS.	Blood samples collected before and post space missions were exposed to ground-based X-rays.	Chromosome aberration frequency	Yes
Lu et al.^50^	ISS Expedition 39	Confluent human fibroblasts (G1 phase) were treated with bleomycin in space.	γ-H2AX and gene expression	No

* Synergy in terms of effect of microgravity on the repair of artificially induced DNA damage

A review on data about heavy ion radiobiology or health risks caused by exposure to space radiation, or the effect of radiation and/or microgravity at the tissue level (bone/muscle loss) is beyond the scope of this paper.

## Simulated microgravity studies

### DNA damage induced by simulated microgravity

As opportunities for true spaceflight experiments are rare, various studies have been conducted using ground-based devices that simulate certain aspects of microgravity. Common analogs for microgravity include rotating wall vessels (RWV), and 2D and 3D clinostats for cultured cells,^11^ and the hind limb suspension model for rodents.^12^ Other approaches, such as airplanes flying in a parabolic pattern or free drop towers also offer true microgravity for a short duration.^11^ For humans, the bed rest model is used to simulate the effects of microgravity on various physiological systems, especially for studies of bone, muscle and the cardiovascular system.^10^

Using these microgravity analogs, researchers have reported a range of data concerning the induction of DNA damage by simulated microgravity. For instance, simulated microgravity has been reported to induce DNA single strand breaks in human retinal pigment epithelial cells (hRPE) cultured in RWV. At 48 h after returning to the 1 g gravity condition, this DNA damage persisted and the production of the inflammatory marker prostaglandin E2 (PGE2) increased. However, hRPE cells previously treated with the anti-inflammatory agent cysteine showed less DNA damage and no PGE2 release.^13^ The bed rest model for studying the effect of microgravity on human physiology has also revealed an increase of 8-oxo-7,8 dihydro-2′ deoxyguanosine (8-oxo-HdG), which is considered a marker of oxidative DNA damage.^14^ Taken together, these results from cell models and human subjects suggest that various forms of ground-based microgravity analogs may indeed induce oxidative DNA damage. Many studies that reported measurements of simulated microgravity-induced reactive oxygen species (ROS), but not specific DNA damage markers, are not included here. Simulated microgravity alone does not appear to induce double strand breaks (DSB) in cells with normal genetic background. However, DSBs were found in mouse embryonic stem cells that were deficient in DDR and cultured under RWV,^15^ suggesting that perhaps some DSB type damage is induced by simulated microgravity, but is typically repaired in DDR competent organisms.

### Effects of simulated microgravity on DDR

In addition to the aforementioned DNA damage, there is evidence indicating that key elements of the DDR machinery are also affected under simulated microgravity alone.^16–18^ For instance, DNA damage, as well as decreased expression of DNA repair genes involved in mismatch repair, base excision repair, nucleotide excision repair, and the down regulation of p53, was observed in proliferating lymphocytes grown in simulated microgravity.^17^ Furthermore, p21 up regulation occurred rapidly in lymphocytes exposed to real microgravity from parabolic flights and under simulated microgravity in 2D clinostats, suggesting a p53-independent mechanism.^19^ Additionally, the activity of the DNA strand break sensor poly (ADP-ribose) polymerase 1 significantly increased under simulated microgravity.^20, 21^ Further studies on mouse embryonic stem cells cultured in a 3D clinostat showed that simulated microgravity alone did not induce DNA damage, but it did affect radiation-induced DNA repair.^22^

Most of the studies on the effects of simulated microgravity on the DDR have used low-linear energy transfer (LET) X-rays or γ rays to generate high levels of DNA damage. These damaged cells were then allowed to repair under different gravity conditions. However, our review of the studies performed under this experimental design has revealed conflicting results. For example, human lymphocytes exposed to 1.5 Gy of X-rays and cultured in a clinostat showed a higher number of X-ray induced chromosome aberrations in comparison to control cells cultured under the static 1 g condition.^23^ But, researchers using the NASA-designed RWV bioreactor found no significant difference in high energy proton radiation-induced (60 MeV protons or 250kVp X-rays in the dose ranges of 0–6 Gy) chromosome aberrations between human lymphocytes cultured in normal vs. simulated microgravity conditions, indicating that DNA repair was not affected.^24^ Even so, more studies do suggest that simulated microgravity has an effect on DDR. Specifically, in lymphoblastoid TK6 cells irradiated with γ rays and incubated for 24 h in a simulated microgravity environment, a significant reduction in apoptotic cells, increased number of cells in G1-phase, and higher frequencies of micronucleated cells and mutations were reported in comparison to cells that were exposed to the same doses of radiation while maintained in 1 g.^25^ Greater mutant frequency was also found in human lymphocytes after ionizing radiation exposure in simulated microgravity.^26^ In both TK6 cells and peripheral blood mononuclear cells (PBMCs), the increase in mutation rate was associated with a reduced rate of radiation-induced apoptosis, again indicating a DDR change. Meanwhile, Omics studies such as transcriptome and microRNome analysis have provided evidence that simulated microgravity affects the DDR to ionizing radiation in human PBMCs by causing downregulation of DDR pathways associated miRNAs like miR-7 and miR-27a.^27^ Ultimately, simulated microgravity delayed DNA repair of radiation-induced DSBs in human lymphocytes, and as a consequence, the genotoxic effects of ionizing radiation increased.^28^ It should be noted, however, that few studies have employed high-LET charged particles to induce DNA damage under simulated microgravity conditions. In one such study, simulated microgravity was shown to decrease carbon ion radiation-induced cell survival and increase apoptosis in human B lymphoblasts. Such effects of simulated microgravity were associated with increased heavy ion radiation-induced intracellular ROS generation.^29^ In another study, increased apoptosis and DNA damage were found in the sperm of mice that was exposed simultaneously to carbon ions and simulated microgravity (Hindlimb suspension).^30^ Together, these results suggest that, in most reported studies, simulated microgravity alters the expression of genes involved in DDR, and indeed affect the cellular response to radiation-induced damage (Table 1). More work is needed, however, to better understand the effects of microgravity on cellular repair processes in response to high LET radiation-induced DNA damage.

## Spaceflight studies

### DNA damage from spaceflight

In contrast to the large number of studies that have been conducted on the ground using particles generated in accelerators, results describing DNA damage from direct exposure to natural space radiation are few. Detection of direct biological damage by space radiation is challenging not only due to the low dose and the low dose rate nature of the space environment, but also due to the possible synergistic effects of microgravity. In one such attempt, fixed human cervical carcinoma (HeLa) cells were flown in the Russian MIR space station for 40 days or on the Space Shuttle for 9 days. The resulting DNA damage levels, as measured by enzymatic incorporation of [3 H]-dATP from terminal deoxyribo-nucleotidyl transferase, correlated with space flight duration,^31^ suggesting that the measured DNA damage was caused by space radiation and was dependent on the length of the space flight. In another experiment, human lymphoblastoid TK6 cells were sent to the International Space Station (ISS) and kept frozen in space for 134 days so that the damage could accumulate (while the impact of microgravity was simultaneously minimized). The cells flown to space showed an increase in thymidine kinase deficient (TK(-)) mutations over the ground controls.^32^ In a similar study, frozen TK6 cells were also analyzed for DSBs by measuring the phosphorylation of the histone H2AX (γ-H2AX). The induced DSBs appeared as a dense track of ionizations and excitations along the particle path.^33^ Similarly, during the Foton-M3 Mission (total 12 days), normal human dermal fibroblasts fixed after 4 days in orbit were found to have increased DSBs.^34^ The γ-H2AX assay for detecting DSBs has also been performed on human fibroblasts cultured at 37 °C on the ISS for 14 days. Although the average number of γ-H2AX foci was similar between the flight samples and ground controls, the flown cells exhibited several track shaped foci that were similar to those induced by high-LET space radiation analogs on the ground.^35^ Together, these results suggest that DNA damage can be directly attributed to the space radiation environment.

Another effect attributed to space radiation is the induction of chromosome aberrations in white blood cells of astronauts after returning from space.^36^ Such damage can be observed only after a 3–6 month duration mission, but not a 2-week shuttle mission.^36^ Similarly, analysis of chromosomal aberrations in blood cells from one Italian and eight Russian cosmonauts were analyzed following missions of different duration on the MIR space station and the ISS. Although an increase in chromosome damage was observed in some cases, the authors did not detect a correlation between flight history and chromosome damage.^37^

DNA damage induced by spaceflight has also been detected using other biomarkers. At times, however, it has been difficult to properly isolate the effects of space radiation from other space environmental factors. For instance, urine samples collected from astronauts have been analyzed for oxidative DNA damage by measuring 8-hydroxy-2′-deoxyguanosine (8-OHdG) before, during, and after spaceflight missions. 8-OHdG excretion was unchanged during spaceflight but increased after flight.^38^ The same study found no changes in 8-OHdG excretion either during or after a short-duration spaceflight,^38^ suggesting that elevated 8-OHdG excretion may depend on the length of the mission. Even though astronauts were exposed to space radiation during the mission, the radiation level was so low that the oxidative damage was perhaps caused by microgravity in LEO or hypergravity experienced during re-entry. Due to the limited in vivo space-based studies, and other factors that may contribute to 8-OHdG changes, it is evident that further work is needed to understand the increased 8-OHdG measured in astronauts.

### Effects of spaceflight on DDR

Potential effects of spaceflight on the DDR have been investigated since the early days of the human space program. Experiments aimed at addressing such effects have been conducted according to several experimental scenarios: (i) pre-flight induction of high levels of DNA damage with radiation prior to launch, (ii) exposing samples to short-ranged particles in space, or (iii) exposing samples to radiation shortly after landing. In these studies, the combined spaceflight factors include not only microgravity and the background space radiation, but also factors, such as hypergravity experienced during launch or re-entry. The experiments carried out in the Gemini III and the Gemini XI manned spaceflight missions were designed to test synergistic effects between ionizing radiation and other stress factors associated with spaceflight. During these missions, cultured human lymphocytes were exposed to ^32^Pb particles in space after reaching the microgravity condition. Chromosomes analysis after Gemini III mission showed an increased frequency of β-induced deletions compared to the ground controls, whereas the frequencies of dicentrics and rings were similar. However, the synergistic effect observed after Gemini III could not be confirmed after Gemini XI.^39, 40^ Early studies on the combined effects of microgravity and radiation have been reviewed previously by Horneck, and by Keifer and Pross, who concluded that spaceflight does affect the development of organisms, but argued that the majority of spaceflight experiments showed little effects of microgravity on the repair of radiation-induced DNA damage.^41, 42^ More recently, attempts at understanding the combined effects of space radiation and microgravity were implemented during the STS-91 Shuttle mission. In the Shuttle experiment, researchers showed no effect of microgravity on either the biochemical reactions involved in DNA damage repair by T4 ligase^43^ or on the repair and replication carried out by Taq polymerase and Polymerase III in response to chemically induced DNA damage.^44^ Beyond these in vitro enzymatic studies, experiments involving bacteria and yeast have provided additional results. During the Spacelab mission IML-2, frozen *Escherichia coli* and *Bacillus subtilis* bacteria were exposed to X-ray and ultraviolet (UV) irradiation before launch. Once in Spacelab, the bacteria were thawed for up to 4.5 h and frozen again until landing, and then assessed for DNA repair. Ultimately, no significant differences were found either in the re-joining of DNA strand breaks or in the survival curve between microgravity conditions and 1 g controls, suggesting that cells were able to repair radiation-induced DNA damage under real, albeit brief, microgravity.^45^ In another study (STS-84), the temperature dependent repair mutant rad54–3 of *Saccharomyces cerevisiae* yeast was irradiated with^46^ Ni β particles and allowed time to repair under different temperatures. By measuring the amount of remaining, unrepaired breaks, researchers demonstrated that there was no difference in double-strand break repair between the flight and ground control samples, suggesting no significant impact of the real microgravity condition on this process.^47^ While these studies on bacteria and yeast did not show impaired DNA repair in microgravity, studies on more complex organisms and human cells have yielded very different results. For instance, a study on *Caenorhabditis elegans* showed that several DDR genes were differentially expressed during the 16.5-day Shenzhou-8 space mission, suggesting possible enhanced DDR under microgravity.^48^ Overall, most of these studies suggest no effect of space flight on the DDR (Table 2).

Further studies in space have also been conducted using bleomycin, a chemotherapy drug that is known to induce DNA damage, including DSBs. It has been reported that human colon carcinoma cells (HCT-116) treated with bleomycin for 2 days in space (Space Shuttle STS-95) showed no difference in frequencies of microsatellite mutations when compared to the ground controls.^49^ In a more recent experiment, confluent human fibroblasts, which were arrested in the G1 phase of the cell cycle, were also treated with bleomycin on the ISS and fixed after 3 h. In this study, analysis of bleomycin-induced DSBs and gene expression changes showed no significant difference between the flight and ground-treated samples, indicating a lack of microgravity effect.^50^

Another experimental design compares how human blood cells respond to ground-based artificially induced DNA damage before and after a space mission. In one study, blood was collected from an astronaut before flight and within 24 h after the 9-day STS-103 mission. After collection, both samples were exposed to γ rays of doses between 0 and 3 Gy. Comparison of the dose response for total chromosomal exchanges showed no differences between pre-flight and post-flight samples suggesting that microgravity had no lasting effect on DNA repair.^51^ Alternatively, Greco and colleagues reported an enhancement of approximately 1.2–2.8-fold in the chromosome aberration frequency in a post-flight cosmonaut blood sample compared to parallel pre-flight data after exposure to ground based X-rays.^37^ Interestingly however, for cosmonauts involved in more than one space flight, the amount of chromosomal aberrations was in the range of the background before the mission started and did not depend on the total duration of flights.^52^ Whether exposure to space radiation during prior space missions caused this effect by impacting the response to damages incurred in subsequent missions has been investigated in astronauts as well. Analysis of chromosome damage in blood lymphocytes collected from five astronauts before and after their first and second long duration space flights detected an increase in chromosome aberrations after both flights, with no significant impact of prior space travel.^53^ Although these results are far from conclusive, taken together, studies from a variety of model systems and humans subjects suggest that spaceflight may indeed play a role in altering the DDR to radiation-induced damage. Further work is needed in the true space environment to determine the exact magnitude and characteristics of such an effect, and understand its impact on human health.

When faced with DNA damage, programmed cell death is an important component of the DDR. Therefore, in addition to the studies aimed at detecting DNA damage levels, investigators have also analyzed endpoints, such as apoptosis. Spaceflight studies conducted on human lymphocytes have shown an increase in apoptosis-related markers, such as DNA fragmentation, cleaved-poly (ADP-ribose) polymerase, and elevated mRNA levels of p53 and calpain after 48 h on board the ISS.^54^ Furthermore, cell cycle-related genes, such as p21, were inhibited in the muscle of rats from space flights compared to rats on the ground.^55^ Such studies indicate that spaceflight factors may indeed influence the rate of apoptosis and/or cell cycle regulation.

## Conclusions

Space environmental factors can cause damage to the DNA, resulting in potential detrimental health consequences. Despite the low dose and dose rate nature of space radiation, there is evidence suggesting that cosmic rays induce DNA damage both in cultured cells and in astronauts’ blood cells. There is also evidence indicating that space environment can generate oxidative DNA damage in vivo. For instance, as mentioned above, 8-OHdG was found in urine of astronauts after flight.^38^

Ultimately, though the biological outcomes of any spaceflight-induced DNA damage also depend on the cellular capacity for repair. However, results from ground and spaceflight studies concerning microgravity’s effects on the DDR have been conflicting. While many studies have reported increased sensitivity to radiation and decreased DNA repair under simulated microgravity (Table 1), most of the experiments conducted in space have shown no effects of spaceflight on the capacity of the cells to repair the artificially induce DNA damage (Table 2), both within and across model systems. In some cases, studies that examined the same endpoints reached opposing conclusions. Perhaps this stems from the fact that ground analogs for microgravity are known to produce some, but not all of the biological effects observed in the true space environment. Experiments conducted in parallel in space and on the ground are needed to fully justify the use of ground-based microgravity analogs.

DNA damage and DDR in space are a complex issue. Although the present review focuses on the interplay of microgravity and space radiation, other environmental factors faced during space travel can also induce DNA damage and influence the DDR. For example, when inhaled, dust particles covering the surface of celestial objects^56, 57^ or toxic chemicals could lead to ROS production and therefore generate DNA damage. Furthermore, elevated CO_2_ present in spacecraft^58^ should be considered as a potential DNA damaging factor.^59^ In addition, inflammatory markers have been reported in astronauts^60^ and it is known that inflammatory mediators can directly affect the DNA repair process by either enhancing or repressing DNA repair. In particular, NF-κB, a key regulator involved in the production of inflammatory proteins, may play an important role in DNA damage sensing and its subsequent repair. Specifically, the DDR directly activates NF-κB, interferon regulatory factors, and a number of ligands for activating immune receptors.^61^ As such, a host of other factors need to be considered and controlled for when conducting and interpreting spaceflight studies.

Furthermore, due to the composition and low fluence nature of GCRs, over a period of several months cells in space are most likely hit by several protons before a high-LET heavy ion transverses them. Thus, prior exposure of cells to the background GCRs might affect the repair of DNA damage induced by protons emitted from a large SPE and vice versa. Indeed, suggestions for this come from studies showing that exposure of human primary cells to protons followed by heavy Ti or Fe ions resulted in a yield of transformation that is greater than the sum of the yields when the cells were independently exposed to protons or heavy particles.^62, 63^ Such synergistic effects were dependent on the sequence and timing between proton and heavy ion irradiation. Similarly, after sequential exposures to protons and Fe ions, human epithelial cells showed the peak of synergistic effects in the induction of chromosome aberrations when the time window between the two exposures was 30 min.^46^ However, another study showed no synergy between proton and Fe ion exposures regardless of the exposure sequence.^64^ Experiments with sequential exposure to protons and Fe ions have also been conducted in animals. Dual exposures of C57BL/6 mice to protons and Fe ions resulted in pronounced molecular alterations in comparison with either source of radiation alone, as measured by a substantial increase in the expression of cytokine IL-13, loss of expression of DNA methyltransferase Dnmt1, and reactivation of LINE-1, SINE B1 retrotransposons, and major and minor satellites.^65^ Finally, exposure of C57BL/6 mice to Fe ions caused an increase in expression of α-smooth muscle cell actin, collagen type III, the inflammatory cell markers mast cell tryptase, CD2 and CD68, the endothelial glycoprotein thrombomodulin, and cleaved caspase 3.^66^ However, exposure to protons 24 h before Fe ions prevented all of the responses to Fe ions,^66^ which again illustrates how the complex composition and timing of space radiation can impact the level of DNA damage and the cellular response to such insults. Therefore, not only microgravity can impact space radiation-induced DDR, but also the radiation types and sequence and the exposure duration may influence the DDR.

In conclusion, carefully designed experiments, perhaps ones implementing a radiation source or other DNA damage agents in space are needed given the conflict results between the reported studies on this topic. Higher levels of DNA damage that is intentionally induced in space would likely be necessary to achieve the results with statistical significance. Meanwhile, experiments using an in-flight 1 g centrifuge compared to the micro-g would provide clues to such questions with damage by natural space radiation levels. However, the assay employed in such studies has to be sensitive enough to detect the DDR to low levels of damage.
